# Irritable bowel syndrome in women: Association between decreased insular subregion volumes and gastrointestinal symptoms

**DOI:** 10.1016/j.nicl.2022.103128

**Published:** 2022-07-28

**Authors:** Nawroz Barazanji, J. Paul Hamilton, Adriane Icenhour, Rozalyn A. Simon, Olga Bednarska, Sofie Tapper, Anders Tisell, Peter Lundberg, Maria Engström, Susanna Walter

**Affiliations:** a Department of Biomedical and Clinical Sciences, Division of Inflammation and Infection, Linköping University, Sweden; b Department of Gastroenterology, Department of Health, Medicine, and Caring Sciences, Linköping University, Linköping, Sweden; c Center for Social and Affective Neuroscience, Department of Biomedical and Clinical Sciences, Linköping University, Linköping, Sweden; d Center for Medical Image Science and Visualization (CMIV), Linköping University, Linköping, Sweden; e Department of Medical Psychology and Medical Sociology, Ruhr University Bochum, Bochum, Germany; f Department of Radiation Physics, and Department of Health, Medicine, and Caring Sciences, Linköping University, Linköping, Sweden; g Department of Health, Medicine, and Caring Sciences, Linköping University, Linköping, Sweden

**Keywords:** AIC, Anterior insular cortex, BPI, Brief pain inventory, dAIC, Dorsal anterior insular cortex, CAT, Computational anatomy toolbox, GBA, Gut-brain axis, GMV, Gray matter volume, HADS, Hospital anxiety and depression scale, HC, Healthy control, IBS, Irritable bowel syndrome, IBS-SSS, IBS severity scoring system, IC, Insular cortex, MDD, Major depressive disorder, MIC, Middle insular cortex, PIC, Posterior insular cortex, qMRS, Quantitative magnetic resonance spectroscopy, ROI, Region of interest, vAIC, Ventral anterior insular cortex, VSI, Visceral sensitivity index, IBS symptoms, Gut-brain axis, Brain morphometry, Gray matter volume, Depression, Insula

## Abstract

**Objective:**

Irritable bowel syndrome (IBS) is a chronic pain disorder characterized by disturbed interactions between the gut and the brain with depression as a common comorbidity. In both IBS and depression, structural brain alterations of the insular cortices, key structures for pain processing and interoception, have been demonstrated but the specificity of these findings remains unclear. We compared the gray matter volume (GMV) of insular cortex (IC) subregions in IBS women and healthy controls (HC) and examined relations to gastrointestinal (GI) symptoms and glutamate + glutamine (Glx) concentrations. We further analyzed GMV of IC subregions in women with major depression (MDD) compared to HC and addressed possible differences between depression, IBS, IBS with depression and HC.

**Design:**

Women with IBS (n = 75), MDD (n = 41) and their respective HC (n = 39 and n = 43) underwent structural brain MRI. IC subregion volumes were estimated using statistical parametric mapping software. General linear model approaches were applied to IC volumetric data and FDR-corrected partial correlation analyses assessed relations between GMV, GI symptoms and Glx concentrations.

**Results:**

IBS patients had significantly smaller IC subregions than HC in both hemispheres but there was no significant difference between MDD compared with IBS and HC for any insular subregion. In IBS, the dorsal anterior insular volumes were negatively correlated with symptoms of nausea and pain, and the left ventral subregion showed a positive correlation with straining to defecate, while the posterior subregion volumes showed no relation to symptoms. In the anterior insula, concentration of Glx showed positive correlations with GMV bilaterally in HC and with GMV of the right anterior insula in IBS.

**Conclusion:**

As the interoceptive cortex, the insula shows substantial and disease-specific structural differences in patients with chronic interoceptive visceral pain. Particularly changes in the anterior proportions might be related to chronic exposure to or enhanced salience towards adverse interoceptive visceral signals and could be linked to biochemical changes, calling for further multimodal and longitudinal work.

## Highlights

• All insular subregions are smaller in IBS compared to healthy women.
• Insular volume associates with GI symptoms independent of psychiatric comorbidity.
• GI symptoms associate with anterior but not posterior insular volume.
• More nausea associated with smaller dorsal anterior insula bilaterally.
• Insula in major depression is not significantly smaller than in healthy women.

## 1 Introduction

Irritable Bowel Syndrome (IBS) is a highly prevalent chronic visceral pain condition affecting approximately 4 % of the population worldwide. IBS has a female predominance (Sperber et al., 2021) and causes a reduction in the quality of life (Simrén et al., 2001) as well as increased health care costs. (Maxion-Bergemann et al., 2006) In addition to pain and altered bowel habits IBS patients often suffer from comorbidities such as depression and anxiety. (Drossman, 2016, Poulsen et al., 2017, Patel et al., 2015, Norlin et al., 2019) In the absence of a biomarker, the diagnosis of IBS relies on symptom criteria. (Longstreth et al., 2006, Drossman and Hasler, 2016) The pathophysiology of IBS is based on a disturbed bidirectional communication between the gut and the brain, and therefore IBS is referred to as a disorder of gut-brain axis (GBA) interactions. (Quigley, 2018) IBS patients commonly suffer from depression and anxiety and there is considerable evidence demonstrating that psychological and gastrointestinal symptoms are interwoven in the complex pathophysiological and clinical picture of IBS. (Midenfjord et al., 2019, Bouchoucha et al., 2013, Addolorato et al., 2008, Midenfjord et al., 2021) Depression is considered an independent risk factor for IBS (Lee et al., 2015) but conversely, disturbances of the GBA can also affect behavior and mood. (Quigley, 2018, Mayer et al., 2015, Chang et al., 2018) In IBS, alterations in the central nervous system have repeatedly been demonstrated. Particularly, both structural and functional alterations of the insular cortex (IC) have consistently been found in relation to chronic visceral pain. (Larsson et al., 2012, Blankstein et al., 2010, Seminowicz et al., 2010, Labus et al., 2014, Grinsvall et al., 2021, Tillisch et al., 2011) Using quantitative magnetic resonance spectroscopy (qMRS), our group could further recently show biochemical alterations in the anterior insular cortex (AIC) in terms of decreased bilateral glutamate + glutamine (Glx) concentrations in IBS patients compared to healthy controls. (Bednarska et al., 2019).

The IC is divided into several subregions delineated both by microstructure and function. (Uddin et al., 2017, Kurth et al., 2010) The AIC is split into dorsal and ventral divisions. The dorsal anterior insular cortex (dAIC) is strongly connected to frontal brain regions and involved in cognitive control while the ventral anterior insular cortex (vAIC) is connected to limbic regions and plays a role in affective processing. (Nomi et al., 2016) The anterior insula has been demonstrated to evaluate the salience of and integrate external and internal stimuli, is a primary hub of the salience network together with the anterior cingulate, (Uddin et al., 2017, Hong et al., 2016) and shows an increased connection to the amygdala in depression. (Kandilarova et al., 2018) The posterior insular cortex (PIC) is connected to sensorimotor brain areas and involved in primary interoceptive processing, with partially overlapping functions to the middle insular cortex (MIC). (Kurth et al., 2010, Nomi et al., 2016) The PIC receives thalamic projections and is activated by numerous nociceptive stimuli thus it is considered as the primary nociceptive cortex. (Craig, 2002) Primary nociceptive information is proposed to function along a posterior–anterior gradient in the IC in which information is processed in the PIC and re-mapped to the AIC to form integrated affective states. (Craig, 2010, Craig, 2009).

Some studies have demonstrated that changes in pain states may induce changes in gray matter volume (GMV). For example, healthy men who underwent daily repetitive painful stimulation for a week had apparent increase in gray matter in the somatosensory cortex contralateral to the site of pain stimulation. (Teutsch et al., 2008) In another study in patients with chronic low back pain, an increase in anterior insula cortical thickness was observed following surgical treatment for reducing pain. (Seminowicz et al., 2011) Brain imaging studies in IBS have consistently demonstrated gray matter alterations of the IC. (Blankstein et al., 2010, Seminowicz et al., 2010, Labus et al., 2014, Grinsvall et al., 2021).

The role of the insula and the salience network has been described in both IBS and major depressive disorder (MDD), but a direct comparison of insular structures in these disorders has never been performed. (Mayer et al., 2015, Manoliu et al., 2014) Interestingly, considerable evidence indicates that the IC is also involved in a broad range of mental disorders, (Evrard et al., 2015) including depression, which is one of the most frequent comorbidities of IBS. In a recent voxel-based morphometry *meta*-analysis, patients with MDD had smaller gray matter volume compared to healthy subjects in the bilateral IC. (Serra-Blasco et al., 2021) IC volume reductions have been linked to several clinical factors such as high pathological guilt in early childhood, (Belden et al., 2015) and increased depression severity and anhedonia. (Sprengelmeyer et al., 2011).

To date, however, there has been only limited investigation regarding the specificity of both volumetric alterations in specific IC subregions and their relation to IBS symptoms as well changes in IBS and MDD. In the present study we compared GMV of insular subregions in independent samples of female IBS and MDD patients and respective healthy controls. We also assessed associations between GMV and gastrointestinal symptoms in IBS. Given available spectroscopy data from a subsample of IBS patients, (Bednarska et al., 2019) we further explored associations between AIC Glx levels and GMV in IBS and healthy controls.

We hypothesized that the GMV of IC subregions in women with IBS would be smaller in comparison to healthy women. Moreover, we hypothesized that insular subregions would be differently affected in IBS and MDD. For example, because of nociception, we expected the primary interoceptive cortex to be altered in IBS but not in MDD. Further we hypothesized that higher gastrointestinal burden would be associated with smaller volumes of IC subregions.

## 2 Materials and methods

### 2.1 Subjects

At the University hospital of Linköping, Sweden, female IBS patients and women with MDD, and their respective healthy control groups were recruited for two independent brain imaging studies. For the first study at the department of Gastroenterology and Hepatology, 75 female patients who met Rome III IBS criteria and 39 healthy women (HC1) without medical history of gastrointestinal symptoms or complaints were recruited by local advertisement. The exclusion criteria were organic gastrointestinal disease, metabolic, neurological, or severe psychiatric disorders. At the adult psychiatric clinic, 41 women diagnosed with MDD with current unipolar depression not explained by another condition were enrolled and 43 healthy women (HC2) were recruited through advertisements to participate in the second study. The Mini-International Neuropsychiatric Interview, (Sheehan et al., 1998) an established clinical tool for DSM-5 ([41]) and ICD-10 (Organization, 2004) psychiatric disorders, was used by qualified interviewers to determine study eligibility. Exclusion criteria were ongoing substance use (except nicotine), psychotic disorder, new antidepressant medication during the month before study participation (two months for fluoxetine), change of the dose of psychotropic medications over the last month (antidepressant and antipsychotic medication) or the last two months (mood stabilizers and anticonvulsants).

General exclusion criteria across both studies were ferromagnetic implants, claustrophobia, and pregnancy. General inclusion criteria were age 18–65; working knowledge of Swedish; willingness and ability to provide informed consent, and ROME III criteria questionnaire for IBS and visceral sensitivity index (VSI) were filled in by all participants. In addition, in the study including IBS patients both a questionnaire battery to assess relevant clinical and psychological measures, and diary data were collected (see below). Both studies were approved by Regional ethical review board (Dnrs. 2013/506–32; 2014/264–32; 2017/17–31). Informed consent was obtained from all participants.

### 2.2 Questionnaires and symptom diary

*Visceral Sensitivity Index (VSI):* A validated questionnaire composed of 15 items that measures gastrointestinal symptom-specific anxiety in terms of cognitive, emotional, and behavioral responses. Sum scores range from 0 to 75 with higher scores indicating more severe GI-specific anxiety. (Labus et al., 2004) *Hospital Anxiety and Depression Scale (HADS)*: The HADS is used to measure symptoms of depression and anxiety. (Zigmond and Snaith, 1983) This scale consists of seven items for both depression (HADS-D) and anxiety subscales (HADS-A), with summed scores on each subscale ranging from 0 to 21; scores of ≥11 indicate clinically significant anxiety or depression. (Bjelland et al., 2002) *IBS Severity Scoring System (IBS-SSS)*: A five item questionnaire that assesses overall IBS symptom severity by evaluating the frequency and the intensity of abdominal pain and distension, the satisfaction with bowel habits, and interference by IBS symptoms with daily life. (Francis et al., 1997) Sum scores indicate mild (75–175), moderate (175–300), or severe (>300) disease with a maximum of 500. In addition, we administered the IBS-SSS extra-intestinal questionnaire (max score 1000) with ten items, four are specific for pain but none of them is specific to abdominal pain. *Brief Pain Inventory (BPI)*: A validated pain assessment tool measuring both the intensity of pain (four items, sensory dimension) and interference of pain with the patient’s life (seven items, reactive dimension). (Cleeland and Ryan, 1994) *Gastrointestinal Symptom Diary (GSD)*: The GSD is a validated diary card in which IBS patients record their gastrointestinal symptoms on 14 consecutive days. (Ragnarsson and Bodemar, 1999) Patients record pain, nausea, stool consistency for every bowel movement (defined by the Bristol Stool Chart, BSC), (Lewis and Heaton, 1997) the presence of urgency, and the need to strain for each bowel movement were reported along a 24-hour time axis. The mean frequency of symptoms per week was computed from the diary data.

### 2.3 Magnetic resonance and regional gray matter volume analysis

**Fig. 1:**
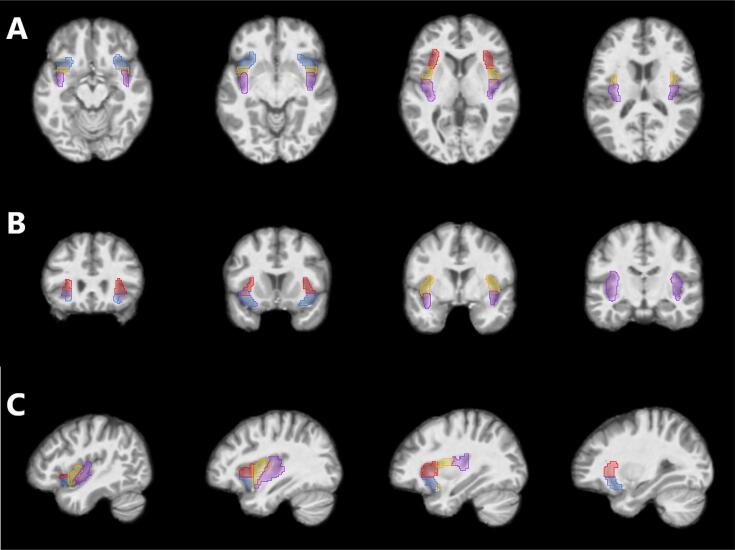
Regions of interest defining the different insular subregions on MRI T1 brain images. A- Axial planes in inferior to superior direction. B- Coronal planes in anterior to posterior direction. C- Sagittal planes of the right hemisphere in lateral to medial direction. Red – dorsal anterior insula; Blue – ventral anterior insula; Yellow – middle insula; Purple – posterior insula. (For interpretation of the references to colour in this figure legend, the reader is referred to the web version of this article.)

MR-images were acquired at the Center for Medical Image Science and Visualization (CMIV) at Linköping University, Sweden. Data from IBS patients and HC1 were acquired on a 3 T Philips Ingenia MRI scanner (Philips Healthcare, Best, The Netherlands) and MDD patients and HC2 were scanned on a 3 T Siemens MRI scanner (Siemens Medical, Erlangen, Germany). Prior to preprocessing, all images were first visually scanned for abnormalities, and one participant in the IBS group with an abnormality indicative of neurological disease was excluded from the analyses. GMV measurement was conducted on the T1 weighted images using the Computational anatomy toolbox CAT12 toolbox (CAT, https://dbm.neuro.uni-jena.de/vbm/) in SPM (SPM12; Wellcome Department of Cognitive Neurology, London, UK) implemented in MATLAB (R2017a, MathWorks Natick, Massachusetts, USA). (Ashburner and Friston, 2000) Each T1 image was reoriented so that all the images would have the anterior commissure as the point of origin. Default settings were used in the CAT12 toolbox. Prior to segmentation, a non-linear deformation field for each image was estimated. Segmentation was done according to a tissue probability image that includes 6 tissue probability classes for: grey matter, white matter, cerebrospinal fluid, bone, non-brain soft tissue, and the background. CAT uses this only for the initial SPM segmentation. For normalization, local optimization is done by affine regularisation (penalising excessive stretching or shrinking) of the aligned images into ‘Montreal neurological institute’ (MNI) space template. Bias reduction done with medium strength of SPM inhomogeneity correction and rough affine preprocessing to reduce problems with deviating anatomy. Spatial registration was done using Diffeomorphic Anatomic Registration Through Exponentiated Lie algebra algorithm (DARTEL) (Ashburner, 2007). The voxel size of the normalized images was set to 1.5x1.5x1.5 mm (Maxion-Bergemann et al., 2006) resolution. Modulation, local enhancement or suppression of a signal, as a function of the deformation, was done by applying, voxel-wise, the Jacobian determinant of the deformation field initially estimated. A quality check was done according to specifications in the CAT12 manual. We examined for artifact and orientation issues one slice for all functional acquisitions within each sample. A check for homogeneity for each group was also performed. The sample correlation matrixes showed generally high correlation between individuals modulated, normalized scans (correlation > 0.84). The regional GMV was then extracted from the segmented gray matter images using insular regions of interest (ROI) provided by A.D Craig (Fig. 1; Supplementary Table 1). (Larsson et al., 2012) GMV for each ROI was extracted using the Multi-image Analysis GUI (MANGO) image processing system (Research Imaging Center, UTHSCSA; https://ric.uthscsa.edu/mango).

In our previous study, single voxel GABA edited qMRS (MEGA-PRESS) was acquired of bilateral AIC, in a subsample of IBS patients and HC1 included herein. In this work, we used the off-resonance-spectra for analysis of Glx concentration in AIC (see Bednarska *et al* (Bednarska et al., 2019) for methodological details). Of note, qMRS data was not obtained for MDD patients and HC2.

For validation purposes, in an additional analysis, we performed an alternative GMV insular parcellation using a different insular mask. For this we used the estimate mean values inside ROI in the CAT-12 toolbox to extract the GMV (in mm^3^) for the anterior (left: 6136 mm (Maxion-Bergemann et al., 2006); right: 6001 mm^3^) and posterior (left: 3203 mm (Maxion-Bergemann et al., 2006); right: 3291 mm^3^) insular parcellations of the Neuromorphometrics atlas (Neuromorphometrics, Inc.).

### 2.4 Statistical analyses

Demographic and clinical variables are reported as means and standard deviations unless indicated otherwise. We performed an independent samples *t*-test for the IBS study questionnaires data with a nonparametric bootstrap procedure with 2000 iterations, and a one-way ANOVA to analyse differences between groups for VSI scores. For analyses within each study, GLM were built to compare IBS and HC1 or MDD and HC2, respectively, for right and left IC and for the insular subregions GMV with total intracranial volume (TIV) and age as covariates of no interest. (Barnes et al., 2010) We further conducted supplementary analysis for IBS vs HC1 with TIV, age, with the addition of anxiety, and depression as covariates.

**Fig. 2:**
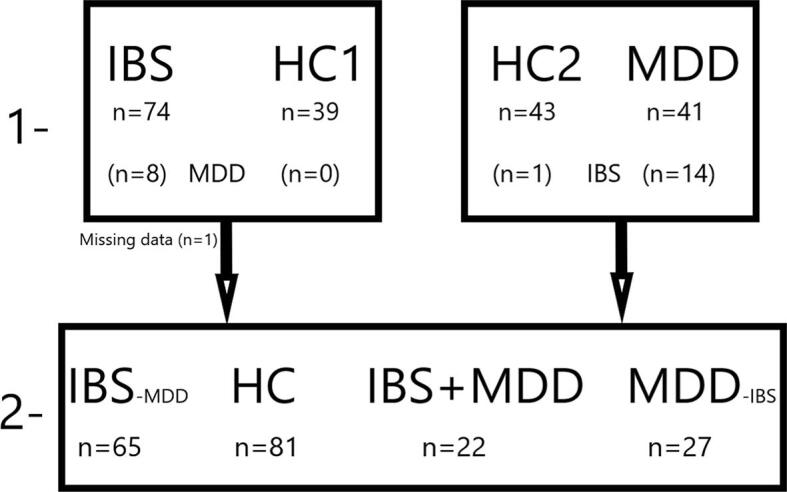
Illustrative chart for inclusion of patients and healthy subjects. Step one is made of two separate general linear models for: IBS vs HC1; MDD vs HC2. For step 2, IBS patients with a score of ≥ 11 in the depression sub-scale of HADS and women with MDD that fulfilled the ROME III criteria for IBS were merged into one group (IBS + MDD) and all healthy women (HC1 + HC2) except for one fulfilling Rome III criteria, were merged into one HC group for the multivariate analysis. IBS: irritable bowel syndrome. HC: healthy control. MDD: major depressive disorder. HADS-D: hospital anxiety and depression scale – depression. For reasons of clarity, we appointed the abbreviation MDD also to IBS patients with HADS-D ≥ 11, i.e. symptom severity indicative of a comorbid clinical depression.

We performed an exploratory comparison of the GMV between IBS, MDD and HC. For this analysis participants in HC2 who fulfilled the Rome III IBS-criteria were excluded (n = 1). MDD patients who fulfilled the Rome III criteria (n = 14) and IBS patients reporting symptoms indicative of comorbid clinical depression based on HADS scores (HADS-D ≥ 11; n = 8) were merged into one group (IBS + MDD); resulting in a sample of IBS_-MDD_, MDD_-IBS_, i.e. without the respective comorbidity, a pooled group of IBS and depression (IBS + MDD), and a pooled HC group (Fig. 2). GMV of the insular subregions were subsequently compared using a GLM with TIV, age, and scanner as covariates to account for possible inter-scanner variability. (Shokouhi et al., 2011, Liu et al., 2020, Biberacher et al., 2016) For all insular subregions, pairwise Bonferroni-corrected between-group analyses were performed. Finally, in a supplementary analysis, we compared the right and left IC subregion volumes of each patient group as a whole, i.e. not excluding patients with possible comorbidities, with the merged group of HC.

To assess possible associations between GMV and gastrointestinal symptoms in IBS patients, partial correlation analyses with a nonparametric bootstrap procedure with 2000 iterations and 95 % confidence interval level were performed. To focus only on gastrointestinal symptoms, anxiety, and depression scores from the HADS questionnaire were added to age and TIV as covariates in the partial correlation analysis to account for possible effects of comorbid psychological symptoms. Further we substituted the covariate TIV with total insular GMV in the partial correlation analyses showing significance in correlation between symptom and insular subregion to test region specificity in symptom association. Glx levels presented in our previous work were used to assess possible association between Glx and GMV in the AIC of HC1 (n = 22) and IBS (n = 33) using a partial correlation analysis with age and TIV as covariates. The significance criterion for all analyses was *p* < 0.05, two tailed, false-discovery rate corrected.

Graphs were created by plotting the residuals rendered from applying multiple linear regression. All statistical analyses were performed using IBM SPSS statistics, version 27 (IBM Corporation, Armonk, NY, USA).

## 3 Results

### 3.1 IBS study

#### 3.1.1 Demographic and clinical variables

The mean age of IBS patients was 32.1 years (±9.2), HC1 33.3 years (±10.9) without significant difference between groups (*p* = 0.51). The following scores are reported as means and standard deviations. Patients presented with high symptom severity (IBS-SSS intestinal: 328 ± 84, IBS-SSS extra-intestinal: 431 ± 185). The number of years from symptom onset was 14.3 years (±11) (n = 39). The HADS-D score was 5.9 (±3.8) and the HADS-A score was 10.3 (±4.2). The BPI-intensity score 16.6 (±9.4), and BPI-interference was 30.8 (±20.2). All group statistics are provided in Supplementary Table 2. Independent sample *t*-tests comparing IBS and HC1 showed significantly higher GI symptom severity (IBS-SSS intestinal *t*_(101)_ = 21.2, *p* < 0.001; IBS-SSS extra-intestinal *t*_(101)_ = 10.6, *p* < 0.001), substantially more symptoms of depression and anxiety (HADS-D *t*_(101)_ = 6.7, *p* < 0.001; HADS-A *t*_(101)_ = 7.6, *p* < 0.001) and increased pain intensity and interferences in patients (BPI intensity *t*_(101)_ = 10.0, *p* < 0.001; BPI-interference *t*_(101)_ = 8.8, *p* < 0.001).

#### 3.1.2 Insula gray matter volumes

**Fig. 3:**
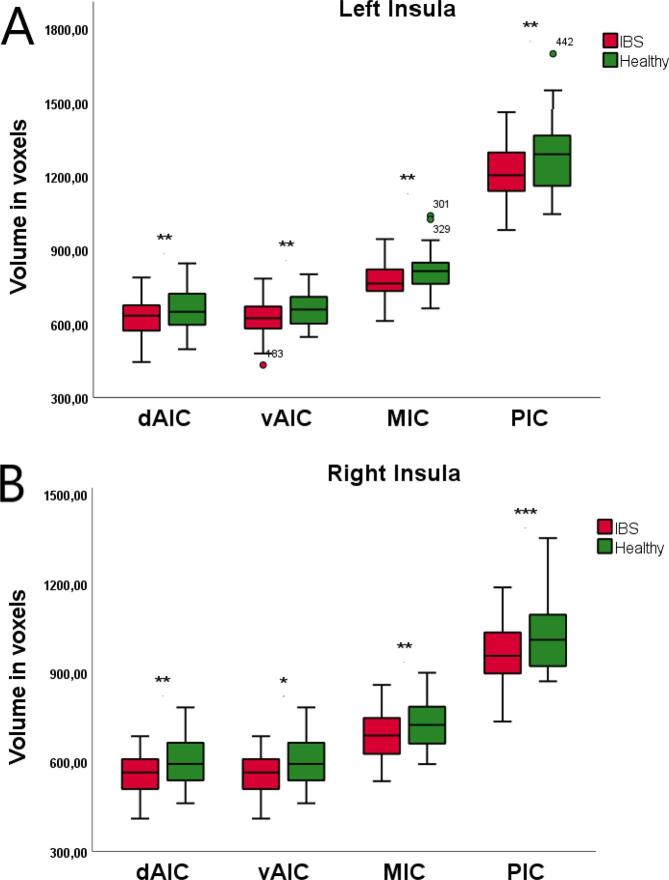
Boxplots with outliers explicitly depicted showing the GMV comparisons of (A) left and (B) right insular subregions between IBS patients (red) and HC1 (green). Error bars represent 95 % confidence intervals. dAIC: dorsal anterior insular cortex. vAIC: ventral anterior insular cortex. MIC: middle insular cortex. PIC: posterior insular cortex. * p < 0.05; ** p < 0.01; *** p < 0.001. (For interpretation of the references to colour in this figure legend, the reader is referred to the web version of this article.)

Multivariate test for GMV for the total IC volumes showed significantly smaller GMV in IBS *F*(_2, 109_) = 4.86; *p* = 0.009; for the left insula *p* = 0.004; and for the right insula *p* = 0.002. Multivariate test for GMV across all subregions showed significantly smaller volumes in IBS for the left *F*_(4, 107)_ = 2.74; *p* = 0.032 and right insula *F*_(4, 107)_ = 3.52; *p* = 0.010. Between-group tests for each of the insular subregions revealed: left dAIC (*p* = 0.008), left vAIC (*p* = 0.002), left MIC (*p* = 0.003), and left PIC (*p* = 0.004) (Fig. 3A); right dAIC (*p* = 0.002), right vAIC (*p* = 0.030), right MIC (*p* = 0.003), and right PIC (*p* < 0.001) (Fig. 3B).

Supplementary analysis with anxiety and depression in addition to TIV and age as covariates showed significantly smaller volumes across all insular subregions (all *p* < 0.05), results are presented in Supplementary Table 3.

Multivariate test of the anterior and posterior Neuromorphometrics atlas insular parcellation showed significantly smaller GMV in IBS in the left insula *F*_(2, 109)_ = 5.50; *p* = 0.005 and right insula *F*_(2, 109)_ = 6.61; *p* = 0.002. Between-group tests for each of the insular subregions revealed: left AIC (*p* = 0.001), left PIC (*p* = 0.004), right AIC (*p* = 0.002), right PIC (*p* < 0.001).

#### 3.1.3 Neuro-behavioral correlations in IBS patients

**Fig. 4:**
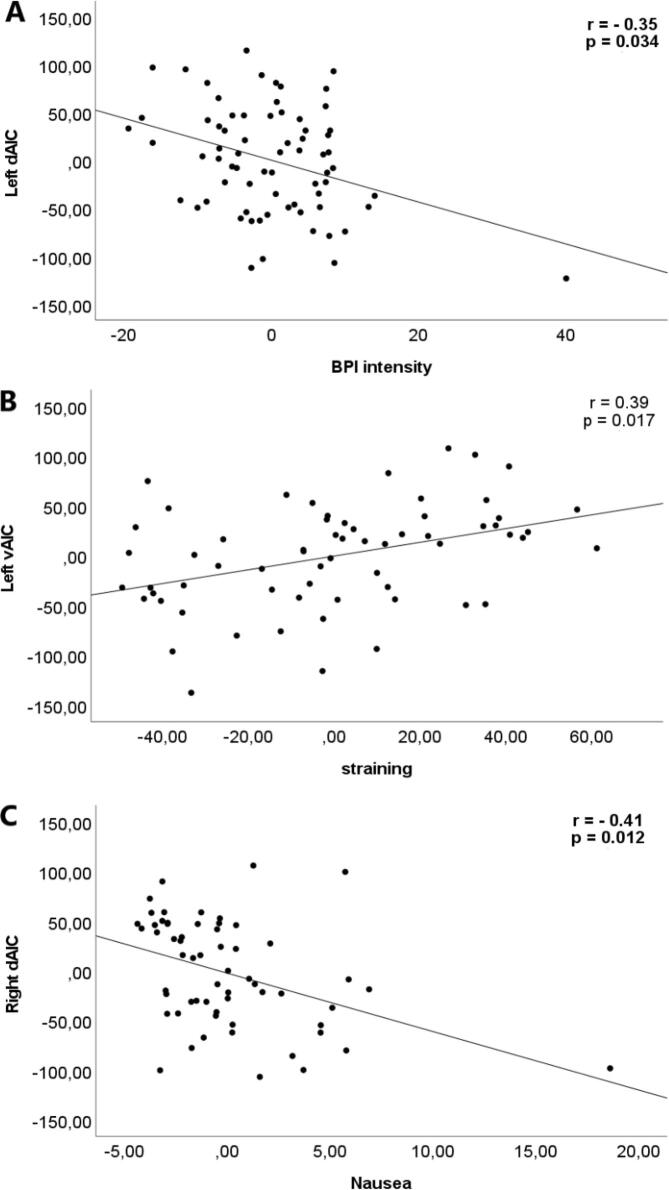
Partial regression plots to illustrate the partial correlations between the gray matter volume of insular subregion in IBS patients and gastrointestinal symptoms. (A) Plotting the residuals of left dorsal anterior insula on residuals of pain intensity. (B) Plotting the residuals of left ventral anterior insula on the residuals of straining. (C) Plotting the residuals of right dorsal anterior insula on the residuals of nausea. Age, total intracranial volume, anxiety, and depression were included as covariates. dAIC: dorsal anterior insular cortex. vAIC: Ventral anterior insular cortex. BPI: brief pain inventory.

***Left insular cortex***. dAIC GMV showed a negative correlation with BPI pain intensity (*r* = -0.35; *p* = 0.034; *CI* = -0.55 – −0.08) (Fig. 4A), and nausea (*r* = -0.39; *p* = 0.013; *CI* = -0.58 – −0.11). vAIC showed a positive correlation to straining (*r* = 0.39; *p* = 0.017; *CI* = 0.17 – 0.59) (Fig. 4B). MIC and PIC showed no significant correlation with clinical symptoms.

***Right insular cortex.*** dAIC showed a negative correlation with nausea (*r* = -0.41; *p* = 0.012; *CI* = -0.63 – −0.12) (Fig. 4C). vAIC, MIC and PIC showed no significant correlation with clinical symptoms.

When performing the partial correlation analysis with the total insular GMV instead of TIV as a covariate, all partial correlations were statistically not significant (p > 0.05) for any insular subregion.

***Years since onset*** of IBS symptoms did not correlate to the GMV of any of the insula subregions.

#### 3.1.4 Glx-GMV analysis

In the subsample of IBS patients for which spectroscopic data was available, the GMV of right AIC showed a significant positive correlation with right Glx concentration (*r* = 0.45; *p* = 0.007), while the GMV of the left AIC showed no significant correlation with the levels of left anterior insular Glx concentration (*r* = -0.12; *p* = 0.51).

In the respective subsample of HC1, the GMV of the right AIC showed a significant positive correlation with the right Glx concentration (*r* = 0.5; *p* = 0.013), and the GMV of the left AIC showed a significant positive correlation with the left Glx concentration (*r* = 0.49; *p* = 0.019).

### 3.2 MDD study

The mean age for MDD patients 37.1 years (±13.7), HC2 35.3 years (±12.8) without significant difference between groups (*p* = 0.51). The multivariate test comparing MDD and HC2 with respect to GMV showed no difference for the total IC *F*_(2, 80)_ = 0.075; *p* = 0.93, nor for the insular subregions in either left *F*_(4, 78)_ = 0.68; *p* = 0.61, or right insula *F*_(4, 78)_ = 1.2; *p* = 0.32 without any statistical significance in the test for between-subjects effect for each of the insular subregions.

Multivariate test of the anterior and posterior Neuromorphometrics atlas insular parcellation showed no difference in GMV between MMD and HC2 neither for left *F*_(2, 80)_ = 0.014; *p* = 0.99, nor right insula *F*_(2, 80)_ = 0.35; *p* = 0.71.

### 3.3 IBS vs MDD analysis

#### 3.3.1 Visceral sensitivity index

ANOVA of mean VSI scores showed significantly higher visceral sensitivity in IBS_-MDD_ patients 43.2 (±14.8) compared with MDD_-IBS_ 14.3 (±11.1), IBS + MDD 33.8 (±19.6) and HC 3.9 (±6.5), *F*_(3, 188)_ = 129.1; *p* < 0.001. Bonferroni corrected *post-hoc* tests for the between-group comparisons of the VSI score were *p* = 0.015 for IBS_-MDD_ vs IBS + MDD and *p* < 0.001 for all other between-group comparisons.

#### 3.3.2 Insular gray matter volumes

**Fig. 5:**
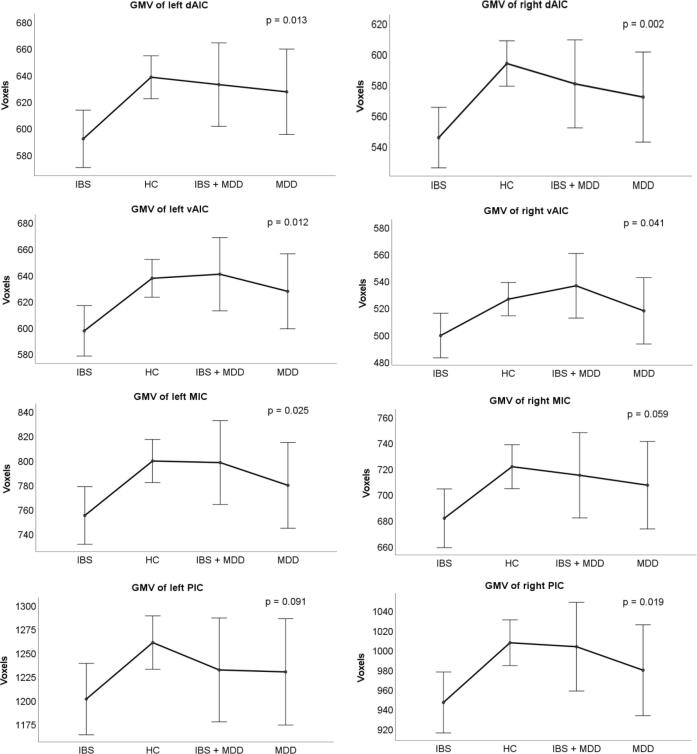
Line chart depicting means with error bars displaying 95 % confidence interval of insular subregion GMV for the four groups (IBS, HC, IBS + MDD, MDD) on x-axis and volume in voxels (1.5x1.5x1.5 mm^3^) on the y-axis. P-values indicate the significance among all four groups for the insular subregion. IBS: Irritable bowel syndrome without depression. HC: Healthy controls. IBS + MDD: Patients with irritable bowel syndrome and depression. MDD: Major depression patients without IBS. dAIC: dorsal anterior insular cortex. vAIC: ventral anterior insular cortex. MIC: middle insular cortex. PIC: posterior insular cortex.

GMV was significantly different among the four groups (IBS_-MDD_, HC, IBS + MDD, MDD_-IBS_) for the multivariate test of both left *F*_(4, 188)_ = 3.21; *p* = 0.014 and right insula *F*_(4, 188)_ = 4.26; *p* = 0.003, with the IBS_-MDD_ group consistently having the smallest IC volumes (Fig. 5). Group differences were attributable to significant differences between IBS_-MDD_ and HC in the IC subregions: left dAIC (*p* = 0.008), left vAIC (*p* = 0.011), left MIC (*p* = 0.029); right dAIC (*p* = 0.002), and right PIC (*p* = 0.021). While no significant differences between the pooled sample of HC and MDD_-IBS_ or IBS + MDD emerged, a tendency towards smaller GMV in participants with MDD compared to HC throughout all insular subregions could be observed. Comparisons between MDD_-IBS_ and IBS_-MDD_ directly did not yield significance either (all *p* > 0.05; descriptive statistics presented in Supplementary Tables 4 and 5). The pooled IBS + MDD disease group showed a tendency toward higher GMV for all insular subregion in comparison to IBS-_MDD_ without reaching statistical significance for any subregion.

Supplementary analyses of the IC subregion volumes for the disease groups as a whole, i.e. not excluding patients with possible comorbidities vs the merged HC (IBS vs HC; MDD vs HC) showed similar results as the analyses with their respective healthy controls, HC1 and HC2, shown in sections 3.1.2 and 3.2 respectively. IBS vs HC, *p* < 0.05 for all IC subregions, and MDD vs HC, *p* > 0.01 for all IC subregions, results are presented in Supplementary Table 6.

Multivariate test using the Neuromorphometrics atlas insular parcellation, among all groups (IBS_-MDD_, HC, IBS + MDD, MDD_-IBS_), was significant for both the left *F*_(3, 189)_ = 4.36; *p* = 0.005 and right insula *F*_(3, 189)_ = 4.36; *p* = 0.005. The Bonferroni corrected pairwise comparison showed significant differences between IBS_-MDD_ and HC in the left AIC (*p* = 0.006), right AIC (p = 0.009), right PIC (p = 0.014). All other between group comparisons were insignificant (*p* > 0.05).

## 4 Discussion

We performed a volumetric analysis of gray matter volumes of insular subregions in women with IBS and healthy controls, and in women with MDD. We found a robust reduction of the total insular cortex volumes and all insular subregions in female IBS patients compared with healthy women. The gray matter volume reduction in IBS was shown using two separate insular parcellations. The study also demonstrated that the volumetric changes in anterior parts of the insula were associated with several of the gastrointestinal symptoms in IBS patients whereas volumes of the middle and posterior parts of the insula did not significantly correlate with any specific symptoms. In addition, we observed a positive correlation between the Glx concentrations and GMV in AIC bilaterally in healthy, and in the right hemisphere in IBS; this is consistent with a gray matter reduction in IBS.

Alterations in GMV, neurochemistry, and functional connectivity have repeatedly been described in chronic pain disorders with key brain areas including the insular cortices, prefrontal cortices, cingulate cortices, and related networks. (Apkarian et al., 2005) In IBS, a majority of research conducted shows a reduction in cortical thickness or GMV in the insula and/or its subregions. (Blankstein et al., 2010, Labus et al., 2014, Grinsvall et al., 2021, Chua et al., 2017, Hong et al., 2014, Jiang et al., 2013) There have also been reports of an increase in the insular gray matter of IBS patients compared to healthy controls. (Zhao et al., 2018) (Piché et al., 2013) Piché *et al*. reported larger PIC in women with IBS and Zhao *et al*. reported higher insular GMV in elderly subjects with IBS compared to healthy; however, both studies were conducted in small IBS samples of<20 patients. In a recent and more extensive study by Grinsvall *et al.*, brain volumetric estimates from 216 women with IBS were compared to those from 138 healthy women. This study found that posterior insula was smaller in IBS but no volumetric differences for the anterior insula were reported. (Grinsvall et al., 2021) In another study Jiang *et al.* reported smaller GMV in posterior, middle, and anterior insula of IBS without subdividing the anterior insula into vAIC and dAIC. (Jiang et al., 2013) In summary, the findings of smaller insula volumes in IBS patients shown in the present study are in line with earlier research (Sheng et al., 2021) and add important new knowledge regarding subdivisions of the anterior portion, the vAIC and dAIC both of which were found to be smaller in IBS, bilaterally. Moreover, we were able to demonstrate that volumetric variability in anterior-mid parts of the insula was associated with several gastrointestinal symptoms, typical for IBS patients, whereas volumes of the mid and posterior parts of the insula did not significantly correlate with any specific symptoms. Importantly, these associations were present despite correction for the effects of anxiety and depression, meaning that the gastrointestinal symptoms but not the psychological comorbidity may be responsible for the volume-symptom relationship.

The mechanisms underlying neuroplastic processes in chronic pain conditions are not fully understood. In one longitudinal study patients surgically treated for chronic back pain leading to pain reduction an increase in anterior insula cortical thickness was observed. (Seminowicz et al., 2011) Another study showed that patients with relapsing painful diseases such as tension headache demonstrate an increase in insular gray matter in pain-free episodes. (Chen et al., 2016).

Disordered neurogenesis has been discussed as one potential mechanism of gray matter reduction in IBS but it is considered less likely since neurons make up a small proportion of the gray matter and are largely outnumbered by glial cells, thus it is likely that neurogenesis accounts for a small portion of gray matter changes measured by MRI. While neurogenesis is still a controversial topic, the astrocytes and oligodendrocyte progenitor cells are known to be able to divide in the adult brain. (Zatorre et al., 2012) Morphological changes in neurons such as changes in number of synapses is another potential mechanism that could explain the gray matter changes like those we report here. (Kleim et al., 2002) Additionally, chronic pain conditions are accompanied by chronic stress and neuroinflammation which could also affect the brain. For example, in patients with chronic back pain, higher serum cortisol levels were associated with smaller hippocampal volume. (Vachon-Presseau et al., 2013) In a primary culture of hippocampal neurons from fetal rat brain, the immunoregulatory cytokine interleukin 2 had a neurotrophic effect on the hippocampal neurons in primary culture, promoting both number and branching of the neurites. (Sarder et al., 1996) Measuring stress levels or cortisol concentrations could therefore be relevant in future studies associating brain measures to gastrointestinal symptoms, and of specific interest in relation to urgency which recently was found to be modifiable by chronic stress. (Icenhour et al., 2020) Another proposed mechanism for volumetric alteration in chronic pain is vascular changes. Periera *et al*., for example, showed that physical activity in mice resulted in an increased blood volume in the dentate gyrus which was associated with different postmortem measures of neurogenesis. (Pereira et al., 2007) In fibromyalgia, brain regions that are smaller in patients than in healthy participants contained equivalent *N*-acetylaspartate (a marker of neuronal viability) concentrations measured by MR-spectroscopy but these regions contained less water than in the healthy participants. (Pomares et al., 2017) In this case it was hypothesized that lower water content in surround tissue may be the result of reduced extravasation due to reduced blood flow. In addition, neurotransmitter dysregulation appears to play a pivotal role in gray matter trophic changes as reviewed by Kang *et al*. who suggest that GABA and glutamate may have a role in microvascular factors that affect local blood flow. (Kang et al., 2019) In IBS, our own group demonstrated that glutamine + glutamate (Glx) concentrations within the IC were lower in IBS than in healthy controls, (Bednarska et al., 2019) suggesting that volume reduction in IBS patients may be associated with an imbalance in neurotransmitter levels in the insula.

### 4.1 Antero-posterior gradient in IBS insular cortex

We demonstrated that volumetric changes in anterior parts of the insula were associated with several key symptoms in IBS, whereas volumes of the mid and posterior parts of the insula did not significantly correlate with any specific symptoms. These findings converge with those from earlier studies showing that the IC function is characterized by an antero-posterior gradient, as well as by interhemispheric differences. (Craig, 2010, Craig, 2009, Craig, 2011).

The PIC has a well-documented network of pathways to primary and secondary somatosensory cortices, to thalamic nuclei, and to mid-cingulate cortex that demonstrate the involvement of the PIC in pain processing. (Lu et al., 2016) The PIC participates in both evoked and chronic pain processing. (Lu et al., 2016) Smaller PIC volume in IBS patients compared to healthy controls has been reported earlier. (Labus et al., 2014, Grinsvall et al., 2021, Chua et al., 2017, Jiang et al., 2013) In our IBS sample, we did not identify any association between symptoms and the GMV of the MIC and PIC. In contrast, Piché *et al*. reported a positive correlation between PIC thickness and the duration of IBS in fourteen females with IBS-D, (Piché et al., 2013) while Jiang *et al*. reported a negative correlation between right MIC thickness and IBS duration as well as anxiety. (Jiang et al., 2013) Elsenbruch *et al*. reported a negative correlation between posterior insular volume and rectal sensitivity in a voxel based morphometry study in healthy men and women. (Elsenbruch et al., 2014) We did not find a correlation between the PIC volume and years since symptom onset for IBS and we did not have a measure for sensitivity and thus we could not replicate the findings of Elsenbruch *et al*. The difference in sample characteristics, analysis method, and brain atlases used may explain the difference in the results. We studied a relatively large number of IBS patients with moderate-to-severe symptoms, and used a robust volumetric measurement; nonetheless, we did not find an association between PIC and pain or other IBS symptoms.

### 4.2 Pain, Nausea, straining and GMV

Left dAIC volume showed a significant negative correlation with pain intensity measured by the BPI questionnaire. Given the AIC’s role in integration of pain, visceral, and emotional signals, these findings indicate that a higher symptom burden is associated with a smaller AIC volume in IBS.

Bilateral dAIC volume was significantly inversely related to nausea in IBS. The right anterior insula is involved in olfaction and visual awareness, and is known to be activated during nausea episodes. (Craig, 2009, Small, 2010, Napadow et al., 2013) The association between nausea and insula function has been described before, recently in a case-control study showing that bilateral insular network connectivity was reduced in nauseated gastroparesis patients compared to healthy controls. (Snodgrass et al., 2020) To our knowledge no one has reported prior to this investigation the association between nausea and insula to be linked to the GMV of the dorsal part of the anterior insula.

IC involvement in pelvic floor muscles and pelvic organs control has been described before, balloon distention of the rectum is usually associated with bilateral insular activation, (Tillisch et al., 2011) another functional-MRI study on healthy subjects showed IC activation during voluntary anal sphincter contraction. (Kern et al., 2004) Left vAIC GMV positively correlated with straining. The ventral part of AIC is known to be involved in awareness and salience. (Seeley, 2019) Straining may reflect the sensation of incomplete evacuation and thereby an urge to evacuate which is an important salient signal.

The addition of total insular GMV as a covariate rendered the correlation analyses statistically insignificant. This could be explained by the fact that all insular subregions are affected in IBS patients, and even though there is a significant association between a certain subregion and symptom, the insular subregions are well interconnected and symptom processing may occur at different levels.

### 4.3 Glx and GMV

In this study we show a lateralized positive correlation between GMV and Glx in right AIC for IBS patients. We did earlier report significantly lower AIC Glx concentrations in IBS compared to healthy bilaterally and a negative correlation between abdominal pain and right insular Glx. (Bednarska et al., 2019) In this study we report a negative correlation for volume of the left AIC and pain scores from the BPI questionnaire that is not specific for abdominal pain. In our research group we demonstrated an increase in activation of the right AIC in expectation of non-aversive stimulus in hypersensitive IBS patients compared to normosensitive. (Larsson et al., 2012) In IBS resting state functional connectivity is increased between bilateral amygdala and the right insula, (Qi et al., 2016) suggesting right insular lateralization in salience and affective pain modulation. The right anterior insula is considered sympathetic, associated with energy consuming negative emotions, and the left anterior insula as a parasympathetic with energy enriching positive and affiliative emotions. (Craig, 2010, Craig, 2009) This may indicate a hemisphere specificity in aversive symptom processing that could explain the lateralized Glx-GMV association.

### 4.4 IBS vs MDD

There was no significant difference in GMV of IC subregions between MDD_-IBS_ and HC or IBS_-MDD_ found, still there was trend towards smaller volumes in all the IC subregions in MDD_-IBS_ when compared to HC and larger volume when compared to IBS_-MDD_. This continuous effect of volume reduction IBS_-MDD_ < MDD_-IBS_ < HC may reflect the somatic load and role of the IC in visceral stimuli processing. Even though excluding the depressed patients from IBS population did not change the findings of the analyses in which IBS patients with depression were compared to HC, and in fact when adding anxiety and depression scores as covariates to that analyses the same results were obtained, this tendency of smaller GMV in all subregions in MDD_-IBS_ supports a role for affective symptoms in insular volume reduction. The IC subregions in the pooled patient group with IBS and depression showed a tendency to be smaller than for those with IBS_-MDD_. An explanation to this could be that this group consisted of substantially more depressed patients, which met Rome III criteria, and in which IBS symptom severity may have likely been much lower than in those patients recruited for our IBS sample, presenting with overall high severity of particularly intestinal symptoms. This difference is also demonstrated in the results of between group differences for VSI where the IBS_-MDD_ patients had significantly higher scores compared to the IBS + MDD group.

Strengths of this study were that the IBS population was relatively homogenous with patients who had long-term GI symptoms. Also, patients had a high symptom burden both in terms of gastrointestinal symptoms but also extraintestinal symptoms which makes the sample representative for many IBS patients. A possible limitation to our study was the lack of male participants, which means that our findings could be limited to females, although, we have taken into consideration that brain morphological measures vary significantly between the sexes.(Jiang et al., 2013, Cosgrove et al., 2007) Glx-measurements included some partial volumes due to the relative size of the acquisition voxels. Moreover, the lack of harmonization between disease groups, that were scanned on adjacent MR-scanners, could also be a potential limitation.

## 5 Conclusion

The decreased gray matter volume of all insular subregions and the difference in IBS symptom association demonstrates the complexity of insular function. This study also suggests that the dorsal and ventral anterior insula are differently involved in the processing of gastrointestinal symptoms. Although affective symptoms may modulate the insular interoceptive signal processing, chronic visceral symptoms independent of depression and anxiety are associated with gray matter volumes in the insula. Longitudinal and preclinical studies are needed to better understand the mechanism behind the observed differences in gray matter volumes.

## Funding

AFA insurance (Dnr: 140407) to SW, ALF, County Council of Östergötland to SW, Knut and Alice Wallenberg Foundation (‘*Seeing Organ Function’*) to PL, Sweden.

## CRediT authorship contribution statement

**Nawroz Barazanji:** Conceptualization, Methodology, Software, Investigation, Writing – original draft, Writing – review & editing, Visualization, Project administration. **J. Paul Hamilton:** Methodology, Investigation, Resources, Writing – review & editing, Supervision. **Adriane Icenhour:** Conceptualization, Writing – review & editing. **Rozalyn A. Simon:** Investigation, Resources, Writing – review & editing. **Olga Bednarska:** Resources, Data curation, Writing – review & editing. **Sofie Tapper:** Methodology, Data curation, Writing – review & editing. **Anders Tisell:** Methodology, Data curation, Writing – review & editing. **Peter Lundberg:** Methodology, Data curation, Writing – review & editing. **Maria Engström:** Writing – review & editing. **Susanna Walter:** Conceptualization, Methodology, Investigation, Resources, Data curation, Writing – review & editing, Supervision, Project administration, Funding acquisition.

## Declaration of Competing Interest

The authors declare that they have no known competing financial interests or personal relationships that could have appeared to influence the work reported in this paper.
