# Supplementary material for: Irritable bowel syndrome in women: Association between decreased insular subregion volumes and gastrointestinal symptoms
Source: Neuroimage Clin. 2022 Jul 28;35:103128. doi: 10.1016/j.nicl.2022.103128 (PMC9421502; doi:10.1016/j.nicl.2022.103128)
Supplement: Supplementary data 1 [file mmc1.docx]

SUPPLEMENTARY

| Subregion | Size |
| --- | --- |
| Left dAIC | 3540 |
| Right dAIC | 3355 |
| Left vAIC | 3567 |
| Right vAIC | 2967 |
| Left MIC | 4040 |
| Right MIC | 3969 |
| Left PIC | 7216 |
| Right PIC | 5025 |

***Supplementary Table 1.*** *Regions of interest (ROI) size in mm^3^ extracted for each insular subregion using ‘Multi-image Analysis GUI’ (MANGO).*

| **Group Statistics IBS - HC1** | | | | |
| --- | --- | --- | --- | --- |
| Score/Group | | N | Mean | Std. Deviation |
| HADS-A | IBS | 73 | 10.3 | 4.2 |
|  | HC1 | 39 | 4.0 | 3.2 |
| HADS-D | IBS | 73 | 5.9 | 3.8 |
|  | HC1 | 39 | 1.5 | 1.8 |
| IBS-SSS - intestinal | IBS | 73 | 328.1 | 83.9 |
|  | HC1 | 38 | 26.4 | 42.6 |
| IBS-SSS extra-intestinal | IBS | 70 | 431.3 | 184.7 |
|  | HC1 | 38 | 86.6 | 78.4 |
| BPI-intensity | IBS | 70 | 16.6 | 9.4 |
|  | HC1 | 38 | 1.1 | 3.0 |
| BPI-Interference | IBS | 69 | 30.8 | 20.2 |
|  | HC1 | 38 | 0.7 | 3.7 |

***Supplementary Table 2.*** *Group statistics for IBS patients and healthy controls (HC1). HADS-A: hospital anxiety and depression scale – anxiety; HADS-D: hospital anxiety and depression scale – depression; IBS-SSS: Irritable bowel syndrome – severity scoring system; BPI: Brief pain inventory.*

| Insular subregion | | A | B |
| --- | --- | --- | --- |
|  |  |  |  |
|  |  |  |  |
| Left | dAIC | 0.008 | 0.007 |
|  | vAIC | 0.002 | 0.011 |
|  | MIC | 0.003 | 0.002 |
|  | PIC | 0.004 | 0.015 |
| Right | dAIC | 0.002 | 0.005 |
|  | vAIC | 0.030 | 0.038 |
|  | MIC | 0.003 | 0.006 |
|  | PIC | 0.001 | 0.001 |

***Supplementary Table 3****. Probability values from general linear models for left and right insular cortex subregions with the p values presented for the gray matter volume of the insular subregion for IBS vs healthy controls 1. The A column represent a model with age and total intracranial volume as covariates and the B column represents a model with age, total intracranial volume, anxiety, and depression as covariates.*

| **Estimates - left insula** | | | |
| --- | --- | --- | --- |
| Subregion/group | | Mean | SEM (95% CI) |
|  |  |  |  |
| dAIC | IBS-MDD | 592 | 11 (570 - 613) |
|  | HC | 638 | 8 (622 - 654) |
|  | IBS + MDD | 632 | 16 (601 - 664) |
|  | MDD-IBS | 627 | 16 (595 - 659) |
| vAIC | IBS-MDD | 597 | 10 (578 - 616) |
|  | HC | 637 | 7 (623 - 651) |
|  | IBS + MDD | 640 | 14 (612 - 668) |
|  | MDD-IBS | 627 | 14 (599 - 656) |
| MIC | IBS-MDD | 755 | 12 (731 - 778) |
|  | HC | 799 | 9 (781 - 817) |
|  | IBS + MDD | 798 | 17 (764 - 832) |
|  | MDD-IBS | 779 | 18 (744 - 814) |
| PIC | IBS-MDD | 1201 | 19 (1163 - 1238) |
|  | HC | 1260 | 14 (1232 - 1288) |
|  | IBS + MDD | 1231 | 28 (1177 - 1286) |
|  | MDD-IBS | 1229 | 28 (1173 - 1285) |

***Supplementary Table 4.*** *Estimates of the mean for the GMV in voxels of the left insular subregions adjusted for the covariates in the model (age, TIV, scanner). SEM: standard error. 95% CI: lower and upper bound for the 95% confidence interval. DAIC: Dorsal anterior insular cortex. VAIC: Ventral anterior insular cortex. MIC: Middle insular cortex. PIC: Posterior insular cortex.*

| **Estimates - right insula** | | | |
| --- | --- | --- | --- |
| Subregion/group | | Mean | SEM (95% CI) |
|  |  |  |  |
| dAIC | IBS-MDD | 545 | 10 (525 - 565) |
|  | HC | 593 | 7 (579 - 608) |
|  | IBS + MDD | 580 | 15 (552 - 609) |
|  | MDD-IBS | 572 | 15 (542 - 601) |
| vAIC | IBS-MDD | 499 | 8 (483 - 516) |
|  | HC | 526 | 6 (514 - 539) |
|  | IBS + MDD | 536 | 12 (512 - 560) |
|  | MDD-IBS | 517 | 12 (493 - 542) |
| MIC | IBS-MDD | 681 | 12 (658 - 704) |
|  | HC | 721 | 9 (704 - 738) |
|  | IBS + MDD | 714 | 17 (681 - 748) |
|  | MDD-IBS | 707 | 17 (673 - 741) |
| PIC | IBS-MDD | 946 | 16 (915 - 977) |
|  | HC | 1007 | 12 (984 - 1030) |
|  | IBS + MDD | 1003 | 23 (958 - 1048) |
|  | MDD-IBS | 979 | 23 (933 - 1025) |

***Supplementary Table 5.*** *Estimates of the mean for the GMV in voxels of the right insular subregions adjusted for the covariates in the model (age, TIV, scanner). SEM: standard error. 95% CI: lower and upper bound for the 95% confidence interval. DAIC: Dorsal anterior insular cortex. VAIC: Ventral anterior insular cortex. MIC: Middle insular cortex. PIC: Posterior insular cortex.*

| Side of insular subregions | Multivariate test | F | Hypothesis df | Error df | p |
| --- | --- | --- | --- | --- | --- |
| Left | IBS vs HC | 2.97 | 4 | 148 | 0.022 |
| Right | IBS vs HC | 3.70 | 4 | 148 | 0.007 |
| Left | MDD v HC | 0.58 | 4 | 115 | 0.675 |
| Right | MDD v HC | 0.90 | 4 | 115 | 0.467 |

***Supplementary Table 6.*** *Results of multivariate tests for IBS females (n=74), healthy controls (HC; n=81), and major depressive disorder females (MDD; n=41). Bonferroni-corrected test for between-group effects of all insular subregions (anterior ventral, anterior dorsal, middle, and posterior) for both left and right insula were all statistically significant (p < 0.05) in IBS vs HC tests, and (p > 0.1) in MDD vs HC tests. df: degrees of freedom.*
